# Association between change in self-reported sugar intake and a sugar biomarker (δ^13^C) in children at increased risk for type 1 diabetes

**DOI:** 10.1017/jns.2020.9

**Published:** 2020-05-11

**Authors:** Martha M. Henze, Elizabeth A. Bemis, Jennifer A. Seifert, Randi K. Johnson, Fran Dong, Marian Rewers, Jill M. Norris

**Affiliations:** 1 Department of Epidemiology, Colorado School of Public Health, University of Colorado Anschutz Medical Campus, Aurora, CO, USA; 2 Department of Pediatrics, The Barbara Davis Center, University of Colorado Anschutz Medical Campus, Aurora, CO, USA

**Keywords:** Added sugars, Dietary biomarkers, Isotopes, Dietary assessment, DAISY, Diabetes Autoimmunity Study in the Young

## Abstract

We examined whether change in added sugar intake is associated with change in δ^13^C, a novel sugar biomarker, in thirty-nine children aged 5–10 years selected from a Colorado (USA) prospective cohort of children at increased risk for type 1 diabetes. Reported added sugar intake via FFQ and δ^13^C in erythrocytes were measured at two time points a median of 2 years apart. Change in added sugar intake was associated with change in the δ^13^C biomarker, where for every 1-g increase in added sugar intake between the two time points, there was an increase in δ^13^C of 0⋅0082 (*P* = 0⋅0053), independent of change in HbA1c and δ^15^N. The δ^13^C biomarker may be used as a measure of compliance in an intervention study of children under the age of 10 years who are at increased risk for type 1 diabetes, in which the goal was to reduce dietary sugar intake.

Consumption of sugars and their role in the development of obesity and related co-morbidities is a major public health concern. Most studies regarding sugar consumption are limited because of their reliance on self-reported intake through FFQ and 24-h dietary recalls. Self-reported intake can be problematic because it can be poorly recalled in terms of the foods consumed and the estimation of portion size and can be subject to social-desirability bias. Moreover, in the context of an intervention study where participants are receiving advice to change their sugar intake, it is important to have a measure of compliance that is not self-reported. Therefore, a biomarker of sugar intake that accurately reflects intake, and can be obtained from a simple blood draw is needed to monitor changes in dietary sugar intake for nutrition research.^(1)^

The carbon stable isotope ratio, ^13^C:^12^C (reported as δ^13^C), is proposed to be an objective measure of sugar intake, as it is naturally elevated in maize and sugar cane relative to other plant-based foods, such that consumption of maize- and cane-derived sugars as well as meat from animals fed primarily on maize (beef cattle, pigs, poultry) would result in an elevated δ^13^C^(2)^. Cross-sectional studies in adults^(3,4)^ and 6- to 18-year-old children^(5)^ have shown that the δ^13^C biomarker is associated with reported added sugar intake, and particularly intake of sugar-sweetened beverages. An intervention in an obese adult population showed that a mean reduction in sugar-sweetened beverage intake (one 12-fluid ounce serving/d (355 ml)) was associated with a reduction of δ^13^C over 18 months^(6)^. Similar findings were reported from 6-month^(7)^ and 12-week^(8)^ interventions focusing on decreasing sugar-sweetened beverage intake in overweight adults.

High reported total sugar intakes^(9)^ and higher glycaemic index of the diet^(10)^ have been associated with increased risk of progression to type 1 diabetes in children. If one were to design a dietary sugar reduction intervention to prevent progression to type 1 diabetes, it would be important to determine whether the δ^13^C biomarker would reflect change in dietary sugar intake in a paediatric population at risk for type 1 diabetes that is typically not overweight.

The purpose of the present study is to determine the relationship between self-reported change in added sugar intake and the δ^13^C biomarker in ‘free-living’ children at increased type 1 diabetes risk between the ages of 5 and 10 years.

## Methods

### Diabetes Autoimmunity Study in the Young (DAISY) cohort

Samples and data included in the present study were previously obtained from the Diabetes Autoimmunity Study in the Young (DAISY), a cohort study with longitudinal follow-up of children who were recruited from the following two populations in Denver, CO, USA: (1) unaffected children with a first-degree relative with type 1 diabetes (*n* 1123); and (2) children from the general population born in 1993–2006 who, through newborn screening for diabetes-susceptibility HLA-DR (human leucocyte antigen-DR) alleles, were identified to have an increased risk for the disease (*n* 1424)^(11)^. The present study was conducted according to the guidelines laid down in the Declaration of Helsinki and all procedures involving human subjects were approved by the Colorado Multiple Institutional Review Board (IRB no. 92-080). Parental consent was obtained for all participants, and assent was obtained from children ≥7 years of age.

Islet autoimmunity was defined as the presence of at least one serum autoantibody against insulin, the tyrosine phosphatase-like protein IA2 (islet antigen 2), GAD (glutamic acid decarboxylase), or Zn transporter 8, twice or more in succession, as described previously^(12)^. Children with islet autoimmunity were followed for the development of type 1 diabetes, as determined by American Diabetes Association criteria. We measured HbA1c using a DCA_2000 Vantage analyser (Siemens Medical Solutions). HbA1c measures the average amount of glucose in the blood over the last 2–3 months by measuring the percentage of Hb with glucose attached. The normal range for HbA1c is between 4 and 5⋅6 %, and levels of 6⋅5 % or higher are indicative of diabetes.

### Dietary assessment

A semi-quantitative FFQ measuring the child's usual dietary intake during the previous year was administered to the parents of the children at each visit. The FFQ has been validated in DAISY using biomarkers^(13,14)^ and 24-h dietary recalls^(15)^. The FFQ contains food items with portion sizes and covers the average consumption frequency with alternatives ranging from: ‘never or less than once a month’ to ‘six or more times per day’. Added sugars (g/d) are defined as sugars and syrups that are added to foods or beverages when they are processed or prepared. Estimates of added sugar intake in our data were based on added sugar values in the United States Department of Agriculture (USDA) Database for the Added Sugars Content of Selected Foods, Release 1, Standard Release 21. Foods not having added sugar values in the USDA database had their added sugar values derived in recipes, using label ingredients, or imputed from similar USDA foods using total sugar ratios. ‘Total sugars’ (g/d) includes added sugars and natural sugars. The sugar-sweetened beverage variable was calculated from the frequencies of reported intake of carbonated and non-carbonated beverages with sugar (soft drinks, punch, lemonade, fruit drinks, iced tea, sports drinks and energy drinks) with one glass, bottle or can (12 fluid ounces) as the standard serving size. Also, 100 % fruit juice was not included as a sugar-sweetened beverage. No dietary advice was offered to the children.

### Study population

From the DAISY population, we selected all children that fulfilled all four of the following criteria: (1) between the ages of 5 and 10 years (inclusive), (2) available dietary added sugar intake data, (3) available HbA1c measures, and (4) an erythrocyte sample available for the measurement of the δ^13^C biomarker on two visits. Of the thirty-nine children who met these four criteria, the two study visits were approximately 2 years apart. The BMI at the first visit was 16⋅9 mg/m^2^. Of the thirty-nine children, thirty (77 %) had islet autoimmunity; none had type 1 diabetes at the time of the included study visits.

### The δ^13^C biomarker

Our primary outcome was the carbon isotope ratio, ^13^C:^12^C (reported as δ^13^C), which can be measured in erythrocytes, plasma, capillary (fingerstick) blood and hair. Given the typical lifespan of erythrocytes of 90–120 d, erythrocyte measures of isotope ratios may better reflect longer-term intake than plasma, and plasma may be better at indicating shorter-term changes in diet^(3,8)^. For the purposes of the present study, we measured isotope ratios in erythrocytes since our dietary intake measure was an annual FFQ and we were comparing change over approximately 2 years. Since levels of δ^13^C may be affected by intake of maize-fed meat, we also measured ^15^N:^14^N, expressed as δ^15^N, which is a biomarker of animal protein/meat intake^(8,16)^, so that this could be accounted for in our analyses. Erythrocytes were sent to the Alaska Stable Isotope Facility at the University of Alaska Fairbanks Water and Environmental Research Center for analysis. Aliquots of erythrocytes were pipetted into tin capsules (Elemental Microanalysis; IsoMass Scientific, Inc.), autoclaved, dried and prepared for isotopic analysis. The tin capsules were crushed and introduced into a Costech Elemental Analyzer (ECS 4010; Costech Analytical Technologies) using an autosampler. The elemental analyser was interfaced to a Delta V Plus isotope ratio mass spectrometer via the Conflo IV interface (Thermo Scientific, Inc.). Isotope ratios are presented in permil (‰) abundance of heavy isotope relative to reference values as follows: δX = (R_sample_ − R_reference_)/(R_reference_) × 1000 (‰), where X is the heavy isotope, R is the ratio of heavy to light isotope (^13^C:^12^C or ^15^N:^14^N), and the reference values are internationally recognised standards calibrated to Vienna Pee Dee Belemnite (^13^C:^12^C = 0⋅01124) and atmospheric nitrogen (^15^N:^14^N_atm-N_ = 0⋅003677). Analytical precision was assessed as the standard deviation of laboratory working standards calibrated to the above reference materials that were measured after every 10th sample; these were typically within 0⋅2‰ for δ^13^C and δ^15^N.

As most living organisms have a smaller ^13^C:^12^C ratio than the Vienna Pee Dee Belemnite carbon standard, the measured δ^13^C values are generally negative. Previous literature demonstrates that there is a positive relationship between δ^13^C and added sugar intake, where greater intake is associated with greater (less negative) δ^13^C levels^(3–5)^. When examining change, one would expect that increasing sugar intake over time, as indicated by a positive (+) change in intake variable, would be associated with increasing δ^13^C, as indicated by a positive (+) change in the δ^13^C variable.

### Statistical analyses

In order to determine whether a change in added sugar intake is associated with a change in δ^13^C over time, we calculated a change variable by subtracting added sugars in the last visit from added sugars in the first visit and similarly for the biomarker. Change variables were also created for total sugar intake, sugar-sweetened beverage intake, HbA1c and δ^15^N. We calculated Pearson correlation coefficients between the intake and biomarker change variables. To evaluate potential covariates, we examined whether change in δ^13^C was associated with sex, race/ethnicity, change in age and change in δ^15^N. Of these, only change in δ^15^N was associated with change in δ^13^C. We used linear regression analysis to test both crude and adjusted models examining the association between change in intake and change in δ^13^C. Even though change in δ^15^N was not associated with the intake change variables (and thus did not meet the classical definition of confounding), we included it in our models because previous studies have shown that the δ^15^N enhances the ability of δ^13^C to predict added sugar intake^(17)^. We performed a sensitivity analysis in the subgroup of children with islet autoimmunity, because this would be the group that would most likely receive a sugar reduction intervention to prevent type 1 diabetes. Given that HbA1c levels increase in children with autoimmunity as they progress to type 1 diabetes^(18)^, we adjusted our analyses for change in HbA1c to examine whether the association between δ^13^C and intake is independent of HbA1c in this population.

## Results and discussion

**Fig. 1.**
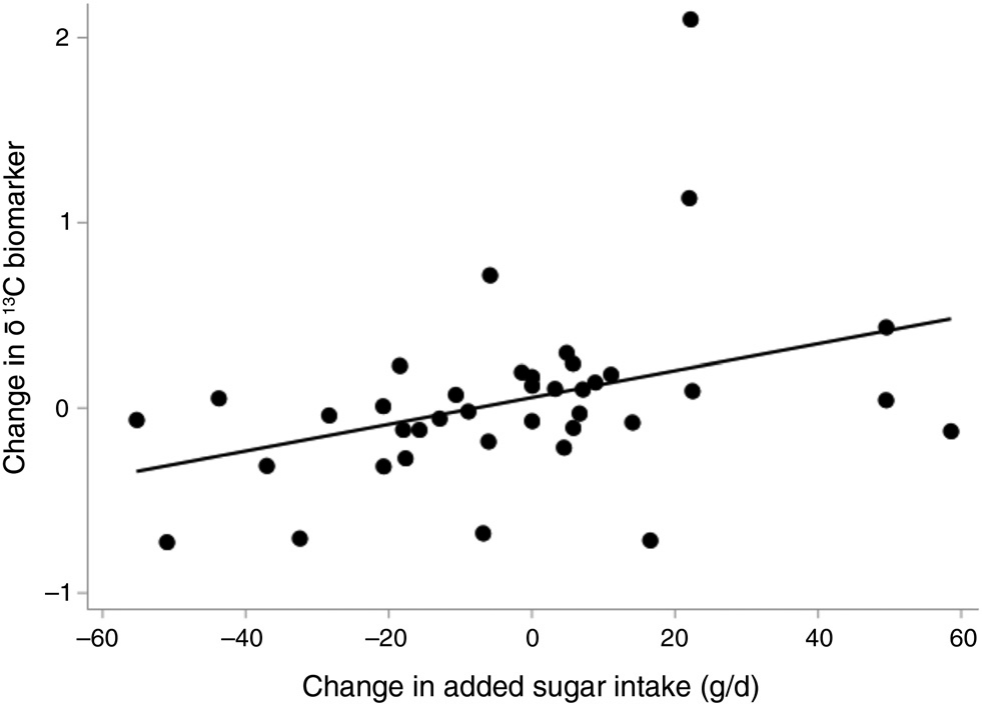
Change in the δ^13^C biomarker by change in reported added sugar intake. Dots represent the data points and the line represents the unadjusted regression line.

Descriptive characteristics of the thirty-nine children in the study are shown in Table 1. The mean age was 7⋅4 years at the first visit and 9⋅4 years at the second visit. Of these thirty-nine children, 79 % were non-Hispanic White. Increasing sugar intake over time, as indicated by a more positive (+) change in intake variable, is correlated with increasing δ^13^C, as indicated by a positive (+) change in the δ^13^C variable (Pearson *r* 0⋅37; *P* = 0⋅02) (Fig. 1).

**Table 1.** Descriptive characteristics of the study populations (*n* 39) at the first and last visits and change between visits (Numbers and percentages; mean values and standard deviations)

	First visit				Last visit		Change between visits	
Variable	*n*	%	Mean	sd	Mean	sd	Mean	sd
Sex, female	19	49			n.a.		n.a.	
Non-Hispanic White*	31	79			n.a.		n.a.	
Have HLA-DR3/4 genotype	6	15			n.a.		n.a.	
Have islet autoimmunity	30	77			n.a.		n.a.	
Age (years)			7⋅39	1⋅09	9⋅36	0⋅70	1⋅96	1⋅02
Added sugars (g/d)			55⋅90	20⋅14	53⋅34	24⋅16	−2⋅55	25⋅03
Total sugars (g/d)			122⋅85	37⋅88	115⋅49	40⋅06	−7⋅37	39⋅54
Sugar-sweetened beverages (servings/d)			0⋅31	0⋅27	0⋅29	0⋅28	−0⋅02	0⋅37
δ^13^C (‰)			−19⋅21	0⋅69	−19⋅17	0⋅66	0⋅04	0⋅49
δ^15^N (‰)			6⋅81	0⋅41	7⋅03	0⋅32	0⋅22	0⋅39
HbA1c (%)			5⋅32	0⋅29	5⋅35	0⋅30	0⋅03	0⋅21

n.a., Not applicable; HLA-DR, human leucocyte antigen-DR.

* The other category included Hispanic American, African American and biracial subjects.

In our unique population of children at increased risk for type 1 diabetes, we found that a change in added and total sugar intake over time was associated with change in the δ^13^C biomarker, independent of change in δ^15^N and change in HbA1c. For every 1-g increase in added sugar intake between the first and the last visit, the δ^13^C biomarker increased by 0⋅0082 (i.e. the biomarker becomes less negative over time) (*P* = 0⋅0053), adjusting for change in δ^15^N and change in HbA1c (Table 2). A similar, although weaker, positive association was seen between change in total sugar intake and change in the δ^13^C biomarker (*β* = 0⋅0029; *P* = 0⋅0497) (Table 2). This is a similar change relationship as that seen in an intervention study in overweight adults^(6)^, indicating that this biomarker may be used to monitor change in sugar intake in children at increased risk for type 1 diabetes. The lack of association between change in sugar-sweetened beverage intake and change in the δ^13^C biomarker (*β* = 0⋅2074; *P* = 0⋅3310) may be due to the low consumption of sugar-sweetened beverages in our relatively young population.

**Table 2.** Associations between change in reported sugar intake and change in the δ^13^C biomarker (*β* Coefficients and 95 % confidence intervals)

	Crude			Adjusted for change in δ^15^N and change in HbA1c		
Reported intake change variable	β*	95 % CI	*P*	β*	95 % CI	*P*
All children (*n* 39)						
Change in added sugar intake	0⋅0029	0⋅0015, 0⋅0129	0⋅0131	0⋅0082	0⋅0024, 0⋅0139	0⋅0053
Change in total sugar intake	0⋅0032	−0⋅0005, 0⋅0070	0⋅0919	0⋅0029	0⋅0000, 0⋅0077	0⋅0497
Change in sugar-sweetened beverage intake	0⋅1798	−0⋅2356, 0⋅5952	0⋅3962	0⋅2074	−0⋅2108, 0⋅6255	0⋅3310
Subset of children with islet autoimmunity (*n* 30)						
Change in added sugar intake	0⋅0080	0⋅0012, 0⋅0147	0⋅0202	0⋅0095	0⋅0029, 0⋅0161	0⋅0046
Change in total sugar intake	0⋅0036	−0⋅0010, 0⋅0082	0⋅1266	0⋅0046	−0⋅0001, 0⋅0093	0⋅0538
Change in sugar-sweetened beverage intake	0⋅1441	−0⋅3482, 0⋅6363	0⋅5662	0⋅1617	−0⋅3232, 0⋅6466	0⋅5133

* Each dietary intake variable was analysed in a separate model.

While a relationship between a change in added sugar intake, particularly in sugar-sweetened beverages, and a change in the δ^13^C biomarker has been reported previously, these studies have been in either overweight^(8)^ or obese^(6,7)^ adults. The only study in children (6–11 years old) reported a cross-sectional analysis^(5)^. Our study extends these findings by showing that change in reported diet is associated with change in the δ^13^C biomarker over an average of 2 years in 5- to 10-year-old children.

The majority of the children in our study had islet autoimmunity, which is a typical population for whom a dietary sugar intervention may be designed, given the epidemiological evidence that higher sugar intake is associated with increased risk of progression from islet autoimmunity to type 1 diabetes^(9,10)^. In the subgroup of thirty children with islet autoimmunity, we found that change in added sugar intake is associated with change in δ^13^C (*β*  = 0⋅0095; *P* = 0⋅0046), while adjusting for change in HbA1c and change in δ^15^N (Table 2). Since HbA1c levels also increase during this time period^(18)^ it is important that we show that the association between change in intake and change in δ^13^C is independent of change in HbA1c.

While erythrocyte δ^13^C provides an assessment of usual diet over several months, plasma δ^13^C may be a better biomarker to use to monitor compliance in shorter-term (<3 month) dietary interventions^(8)^. A limitation of our study is a lack of variability in race/ethnicity, although our study population reflects the race/ethnicity of those at risk for type 1 diabetes. Additional limitations include the relatively small sample size and the reliance on reported diet via a FFQ rather than a more quantified measure of intake, such as food records. And finally, we note that the erythrocyte δ^13^C biomarker is most useful in populations in which the added sugars in the diet is from sugar cane or maize, rather than beets.

### Conclusions

The δ^13^C biomarker was associated with change in reported added sugar intake in young children, suggesting that this biomarker could be used as an objective measure of compliance in an intervention study in which the goal was to reduce sugar intake. This research project has contributed to the body of knowledge surrounding this novel biomarker in providing evidence for the use of this objective measure of sugar intake in children, under the age of 10 years, who are at high risk for developing type 1 diabetes.
